# Development of an interactive e-learning software “Histologie für Mediziner” for medical histology courses and its overall impact on learning outcomes and motivation

**DOI:** 10.3205/zma001328

**Published:** 2020-04-15

**Authors:** Christina Drees, Estifanos Ghebremedhin, Miriam Hansen

**Affiliations:** 1 Goethe University Frankfurt/Main, Dr. Senckenbergische Anatomie, Anatomy I, Clinical Neuroanatomy, Frankfurt/Main, Germany; 2 Goethe University Frankfurt/Main, Educational Psychology, Interdisciplinary College for University Teaching (IKH), Frankfurt/Main, Germany

**Keywords:** E-learning, multimedia, digitalisation, interactive learning, medical education, anatomy and histology

## Abstract

**Objective: **To develop and evaluate an interactive histology learning software for medical students in the preclinical study phase. The educational design of the software was based on current learning theory models, such as the *Cognitive load theory, Cognitive theory of multimedia learning*, and the *ARCS* model, so that the acquired knowledge can be repeated using a diversified design. Moreover, the learning effects achieved by using the software shall be evaluated. Apart from the software’s usability, the influence of the learning theory principles on the students’ motivation shall be assessed.

**Methodology:** The software was evaluated using an experimental wait list control group with a pre-/post-test design (n=213). Depending on the group they were assigned to, students learned the histology contents of chapter “Liver, gall bladder, pancreas” using the traditional program of the Goethe University (n=65), the new interactive software (n=56), or without any of the two software versions (n=92). The influence of the different learning aids on the acquisition of knowledge was assessed with three questionnaires comprising four different multiple choice questions each. For the evaluation of the usability and motivational factors, a second test was added to the questionnaire of both software versions.

**Results:** The interactive software was rated significantly better with regard to usability and motivational aspects than the traditional learning program (*F*(7, 113)=12.48, *p*<.001, partial η^2^=.436). Moreover, use of the interactive software resulted in a significant increase of knowledge acquisition as compared to the group of students who had learned without any of the two software versions (0.77, *p*=.001).

**Conclusion: **With regard to the histology contents, usability was comparable to the official learning program. Interactive elements and the educational design contributed to an increase of the factors that are essential for intrinsic motivation. Thus, our program can be valuable tool to supplement the curriculum as an additional service.

## Zusammenfassung

**Zielsetzung: **Das Ziel der Arbeit bestand in der Entwicklung und der Evaluation einer interaktiven Histologie-Lernsoftware für Studierende der Medizin im vorklinischen Studienabschnitt. Die didaktische Gestaltung der Software orientierte sich an aktuellen lerntheoretischen Modellen wie der *Cognitive Load Theory*, der *Kognitiven Theorie* *multimedialen Lernens* und dem *ARCS*-Modell, so dass das vermittelte Wissen mit Hilfe einer abwechslungsreichen Gestaltung wiederholt werden kann. Weiterhin sollte eine Einschätzung der durch die Software erzielten Lerneffekte erfolgen. Dabei sollte neben der Usability insbesondere der Einfluss lerntheoretischer Prinzipien auf die Motivation der Studierenden beurteilt werden.

**Methodik: **Die Software wurde unter Nutzung einer experimentellen Wartekontrollgruppenstudie mit Prä-/Posttest-Design evaluiert (N=213). Die Studierenden lernten entsprechend ihrer Gruppenzugehörigkeit die histologischen Inhalte des Kapitels „Leber, Gallenblase, Pankreas“ mit dem regulären Programm der Goethe-Universität (N=65), mit der neuen, interaktiven Software (N=56) oder ohne eine der beiden Softwareversionen (N=92). Der Einfluss der verschiedenen Lernmittel auf den Wissenserwerb wurde über drei Fragebogen mit jeweils vier unterschiedlichen Multiple-Choice-Fragen ermittelt. Zur Bewertung der Usability und der motivationalen Faktoren wurden die beiden Softwareversionen über eine Erweiterung des Fragebogens im Rahmen des zweiten Tests evaluiert.

**Ergebnisse: **Die interaktive Software wurde im Hinblick auf Benutzerfreundlichkeit und motivationssteigernde Aspekte signifikant besser bewertet als das bestehende Lernprogramm (*F*(7, 113)=12.48, *p*<.001, partielles η^2^=.436). Weiterhin konnte durch die Verwendung der interaktiven Software eine signifikante Steigerung des Wissenserwerbs im Vergleich zu der Gruppe verzeichnet werden, die ohne eine der beiden Softwareversionen gelernt hatte (0.77, *p*=.001).

**Schlussfolgerung: **Die Anwendung war bezüglich der histologischen Inhalte gleichwertig zum offiziellen Lernprogramm. Durch die Verwendung interaktiver Elemente und das didaktische Design konnte eine Steigerung der für die tätigkeitszentrierte Motivation essentiellen Faktoren erreicht werden. Somit kann unser Programm, besonders unter dem Aspekt der Nutzung als Zusatzangebot, eine wertvolle Ergänzung des Lehrangebots bieten.

## 1. Introduction

### 1.1. Background

Medical histology is one of the basic science courses in the preclinical phase of the medical school curriculum [3], [26]. It provides an important basis for subsequent subjects in the clinical phase of the study, e.g. Pathology [26], [27]. Through changes in the curricula, classroom teaching with histology contents and conventional microscopy has decreased significantly in the past years [8]. Therefore, alternatives such as virtual and digital microscopy as well as interactive platforms play a major role in supplementing curricular lectures [3], [8], [15], [16], [30].

Moreover, by using media-supported teaching and learning alternatives such as the inverted classroom model enables students to actively prepare course content and then deepen their knowledge during the actual classroom session [10].

For the development of suitable digital learning materials it is, however, essential to base the design of the medium on current learning theory models. Thus, educational design options and empirically validated learning theories should already be incorporated in the planning and development phase [5], [12].

Besides a few commercial learning platforms, there are several university histology applications [15], [19], [26], [30], [33]. However, as the study program combines elements like music, voice texts, and interactive quiz questions in a new alternative form, it could be an important addition to existing e-learning services. Moreover, the present study provides insights in the learning success and motivation of medical students that could be useful for the development of future multimedia learning applications.

#### 1.2. Objective

##### 1.2.1. Teaching contents and target group

The aim of this study is the implementation and evaluation of an interactive learning software for medical students in their preclinical study phase.

The purpose of this web-based learning software is to allow for interactive learning and repeating contents of the “Microscopic Anatomy II“ session of the Anatomic Institute of the Johann Wolfgang Goethe University Frankfurt/Main. Course contents comprise the histology of peritoneal and retroperitoneal organs [https://www.uni-frankfurt.de/60716282/Kursus_und_Vorlesung_Anatomie_II, verified on: 16 December 2019]. The educational design is based on the learning theory models and contents of both lecture and practical training and serves as a supplemental multimedia service in addition to the lectures.

##### 1.2.2. Learning theory models

In 1991, Sweller and Chandler developed a model using various approaches to explain the complex process of learning and understanding of new information [4], [32]. The *Cognitive load theory* describes the various causes of cognitive load during the learning process and storage of information. For the development of learning materials this means that the design can influence the cognitive load to a certain extent [4], [32].

According to current interpretations regarding instructional design, the development of learning environments based on cognitivism is divided into various parts [5]. Thus, the learning success can be increased by an educational design in consideration of different principles [5], [21], [22]. The *Cognitive theory of multimedia learning* describes the necessity to combine auditory and visual information in the working memory as a model and link it with information of the long-time memory [21], [22], [24]. Through the implementation of schemes and repetition of learned content, the information can finally be stored in the long-term memory [24]. For this purpose, the presented software contains numerous exercises enabling sustained knowledge acquisition by means of preparation and follow-up of course contents.

In addition to the educational design reported by Clark and Mayer, the *ARCS* model also refers to the motivational aspects that are required for the learning process [13], [14]. The *ARCS* model refers to four superordinate components that shall serve as motivational aspects and was named after the initials of these components: Attention, Relevance, Confidence and Satisfaction shall support endurance in the learning process [13], [14]. Using interactive elements, visual effects and sounds as well as a versatile user interface can help maintain the user’s attention and prevent termination of the learning process [13], [14]. An important feature of the *ARCS* model is the distinction between extrinsic and intrinsic motivation, both of which can be increased with this instruction model [13], [14], [24]. In our software, multimedia and interactive elements like quiz questions with audiovisual feedback, music, direct address and control codes (arrows, display of the terms) were incorporated with free manoeuvrability within the chapter. Besides, the *FEASP* model addresses the emotional elements of the learning process defining fun as an essential factor [2], [24]. For the determination and evaluation of the motivation it is relevant that this is a multi-factorial construct. This can be defined and increased with these models, while the criteria of motivation increase and aspects of usability partly overlap. The concept of usability outlines the user-friendliness of a software evaluating it on the basis of the efficiency and effectiveness of the program as well as learners’ satisfaction [6], [24]. Moreover, further criteria are defined including suitability of the tasks, self-descriptiveness, as well as fault tolerance [7], [24].

#### 1. 3. Questions

The focus was on the comparison of two software versions (the traditional program “Histo-Online” and the new interactive software “Histologie für Mediziner”) addressing the following questions:

1. Does the new, interactive software cover all histology contents of the existing learning program?
2. Are there any differences between the two software versions with regard to knowledge acquisition?
3. Are there any differences between the two software versions with regard to usability?
4. What influence do the learning theories of the new software have on the students’ motivation?

## 2. Methods

### 2.1. Research subject: Comparison of both software versions and group assignment

At the beginning of the study, the participants of the practical training “Microscopic Anatomy II” were divided into two groups. They were randomised to the intervention group “Software interactive” (IG_int) and “Software regular” (IG_reg). According to the group they belong to, participants were asked to learn the histology contents of the chapter “Liver, gall bladder, pancreas” in preparation of the respective training day. IG_int learned the contents using the new software “Histologie für Mediziner”, while IG_reg learned with the existing learning program “Histo-Online” of the Goethe University Frankfurt. Students who claimed to have learned (T2, see below) without the software were assigned to the control group (CG) for the evaluation. Until data collection, the students had two weeks to learn the chapter. The research design provided all students with the opportunity to use both software versions until the end of the practical training. Students could use them as an optional additional offer and the individual use was recorded in the respective evaluation form. The chapter “Liver, gall bladder, pancreas” was used as an example, as the related training day took place after half of the total term. Thus, students had sufficient preparation and learning time between the three tests. Processing time and implemented interactive components correspond to the average processing time and design of the remaining chapters.

#### 2.2. Participants

All study participants (n=213) were in the preclinical phase of their medical education. The mean age was 21.32 years (*SD*=3.8). Table 1 (Tab. 1) shows the group composition.

#### 2.3. Ethics Committee

The project was submitted to the Ethics Committee of the Goethe University. According to the Ethics Committee, their vote is not required. Anonymity of data was guaranteed at all times during the study.

#### 2.4. Material for the learning phase

##### 2.4.1. Learning program “Histo-Online” 

“Histo-Online” was developed by the Dr. Senckenbergische Anatomie of the Medical Faculty of the Goethe University in 2007 and can be used by the students as learning aid and additional service to the lectures and practical training. The relevant learning unit (LU) contains nine different specimens. The information was presented as written texts and histological pictures [https://www.kgu.de/zmorph/histopatho/histo4/pub/index.html, verified on: 16 December 2019]. There are no quiz questions and auditory components like spoken texts or sounds.

##### 2.4.2. Interactive E-learning software “Histologie für Mediziner”

For the preparation of the chapter, eight histological preparations from the collection of the Anatomical Institute of the Goethe University were used. For image processing and graphic design, CorelDRAW X7 (Corel Corporation) was used and the software was developed by means of Adobe Captivate 9 (Abobe Systems Incorporated).

In line with the learning theory models described above, histology contents were presented in spoken form with simultaneous display of the terms. As the software is an additional service for the preparation for the histology exam, static images are used, because static images are used in the exams and a similar design facilitates preparation to the respective exam questions. Control codes (arrows, coloured markings, different magnifications of the histology images) were used to emphasize relevant details and incorporate movements.

In every LU, students have to answer an interactive quiz question after each section. The quiz on the LU contains various types of questions (short answers, sequence, hotspot, drag-and-drop, multiple choice, and true/false tests) with a total of 18 questions on the topic “Liver, gall bladder, pancreas”. After answering a question, they get a direct visual and auditory feedback (demo version: https://tinygu.de/histodemo, verified on: 16 December 2019). Users can control their individual learning speed. LU and quiz are independent of each other. Depending on the personal requirements, individual sections can be controlled individually and repeated as often as desired via the table of contents. Attachment 1 & Attachment 2 show samples of both LU and quiz questions. Attachment 3 contains further information on the development of the software.

##### 2.4.3. Additional learning options

Participants of all groups could attend the histology lectures and learn using the lecture slides and course books. During the course of their study, they completed the mandatory practical training.

#### 2.5. Data collection

##### 2.5.1. Questionnaire

**Figure 1:**
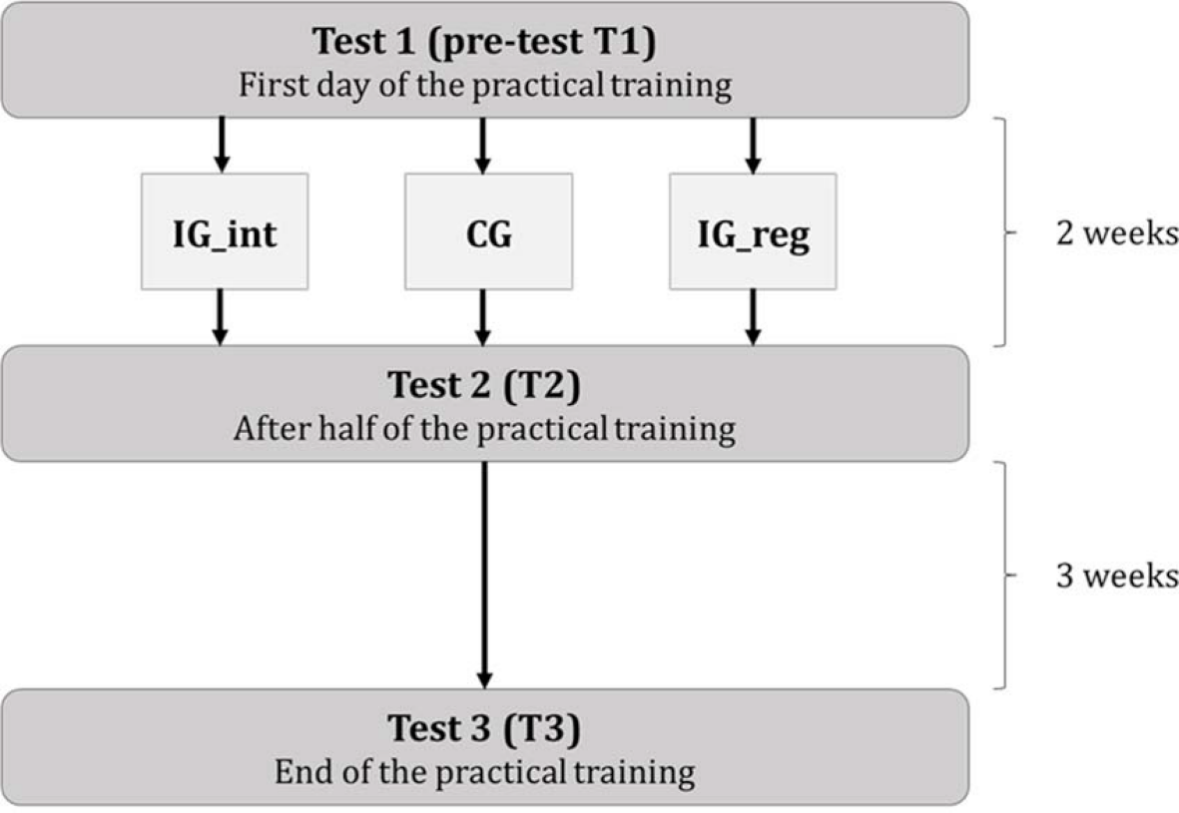
Diagram of the evaluation study. The study was conducted during a mandatory practical training over a period of several weeks. On the first day of the practical training, a pre-test (T1) was performed to assess the participants’ existing knowledge. Thereafter, IG_reg learned with the traditional learning program “Histo-Online” of the Goethe University Frankfurt, while IG_int learned with the interactive software “Histologie für Mediziner” on the OLAT website of the student body of the Medical Faculty of the University Frankfurt. The CG learned without any of the two software versions. All participants could attend the respective lecture and learn using the lecture slides and course books. After two weeks, the changes in the level of knowledge was determined by means of a second test (T2). During this test, usability-relevant and motivational factors were also evaluated. Afterwards, all students had three weeks to learn with both software versions until they had to take a final test (T3).

Learning success was defined and evaluated on the basis of the individual knowledge increase. Data collection took three days. On the first day, a pre-test (T1) was performed to assess the participants’ existing knowledge. Further quantitative data were collected after a study duration of two weeks (T2) and five weeks (T3). All data on the motivational factors and user-friendliness relevant for this study were collected within the scope of T2. T2 provided the opportunity to evaluate the effectiveness of the respective software version independent of the influence of the respective practical training day. Figure 1 (Fig. 1) shows the exact timing of the wait list control study.

All questionnaires were completed by the students in anonymised form and linked to them by means of an anonymised code. Participants were only included in the study, if three questionnaires could be associated to them.

The knowledge acquisition questionnaires contained four different multiple choice questions with five possible answers and one valid answer each. They covered the contents of chapter “Liver, gall bladder, pancreas”. Students had 6 minutes to complete the questionnaires. After completion they entered their answers into the enclosed EvaSys form (Electric Paper).

In addition to the histology questions, questionnaire T2 included seven other items relating to usability and – among other things – visual comfort, clear layout, and subjective knowledge increase, various factors for increasing both intrinsic and extrinsic motivation (see Attachment 4). Moreover, one fundamental criterion of individual motivation was directly evaluated with the item “I enjoyed using the software”. Students had 5 minutes longer to complete questionnaire T2. The additional items were evaluated using an endpoint-based 6 stage Likert scale from 1 (“Strongly disagree”) to 6 (“Strongly agree”).

##### 2.5.2. Data evaluation and analysis

Raw data were entered in Microsoft Office Excel 365 and further analysis performed using IBM SPSS Statistics 25 for Windows. Data on knowledge acquisition were analysed using mixed ANOVA. Usability analysis was performed using MANOVA with subsequent Bonferroni correction.

## 3. Results

### 3.1. Assessment of the learning success

Study participants (n=213) were divided into IG_int (n=56), IG_reg (n=65), and KG (n=92). The preconditions for ANOVA were assessed and confirmed.

A statistically significant main effect time was observed (sphericity assumed: *F*(1, 210)=86.36, *p*<.001, partial η^2^=.291) with higher values at the second point of measurement (*M_T1_*=0.94, *SD_T1_*=0.85, *M_T2_*=1.83, *SD_T2_*=1.25). Moreover, a significant main effect of the group was observed (*F*(2, 210)=4.16, *p*=.017, partial η^2^=.038).

**Figure 2:**
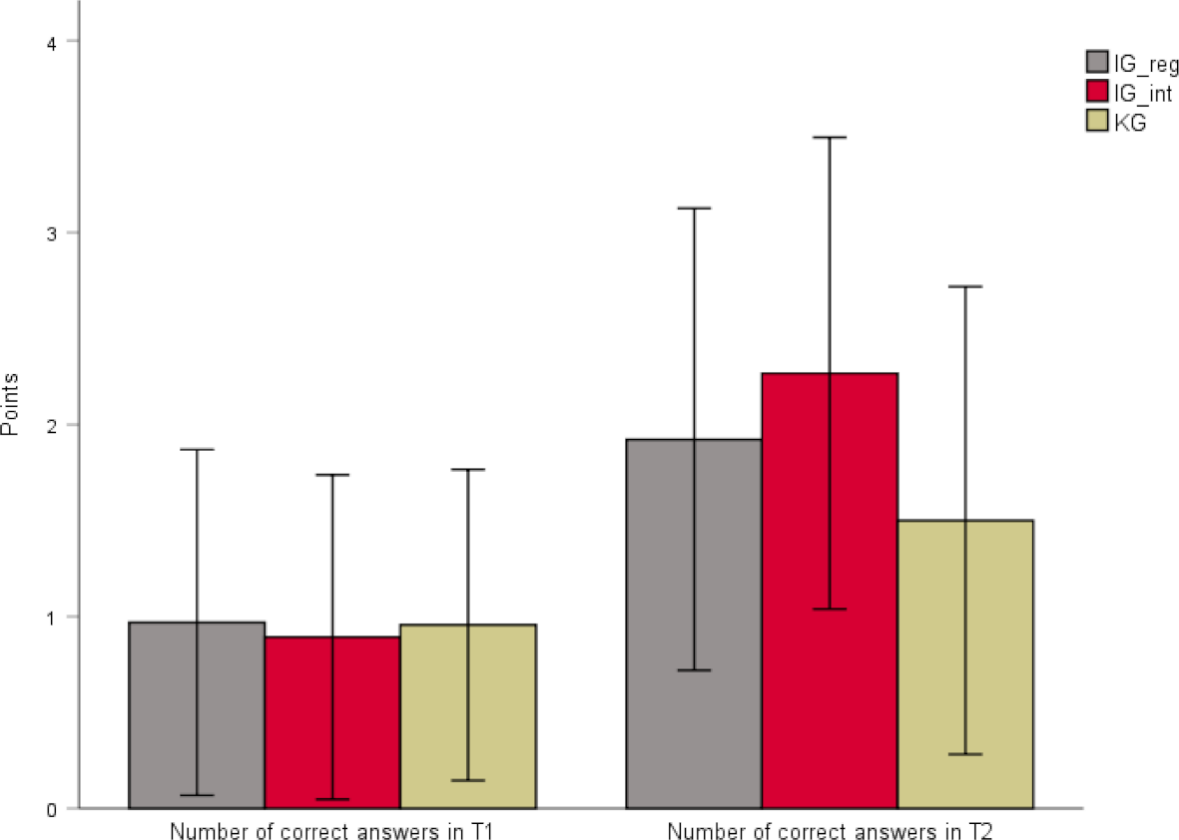
Results of the evaluation of the learning success. Participants (n=213) were assigned to either IG_reg (n=65), IG_int (n=56), or CG (n=92). The number of correct answers in the pretest (T1) at the beginning of the practical training and the second test (T2) after two weeks was measured. In every test, a maximum of four points could be achieved. Inductive statistics used are outlined in 3.1. Tab. 2 shows the exact mean values and standard deviations.

There was also a significant interaction effect between time and the various study groups (sphericity assumed: *F*(2, 210)=5.65, *p*=.004, partial η^2^=.051). After a study duration of two weeks, the number of correct answers varied significantly between the groups (*p*=.001). Tukey’s HSD tests demonstrated that the number of correct answers was significantly higher in group IG_int due to the use of the interactive software as compared to the CG (0.77, *p*=.001). This item was not significantly different in the other groups (see figure 2 (Fig. 2) and table 2 (Tab. 2)).

All groups had the opportunity to use both software versions as learning aid between T2 and T3. This led to a knowledge increase in all groups. Within the groups who used the interactive software as an additional service, a trend towards a higher increase was observed as compared to the group who learned without the interactive software. Regarding the learning success, it was not significant whether the interactive software was used at an earlier or later stage. Due to the smaller group size of the subgroups between T2 and T3, data are displayed using descriptive statistics (see table 3 (Tab. 3)).

#### 3.2. Determination of motivational factors and usability

Participants of the IG_int (n=56) and IG_reg (n=65) group were asked to evaluate the learning motivation and usability. The preconditions for MANOVA were assessed and confirmed.

**Figure 3:**
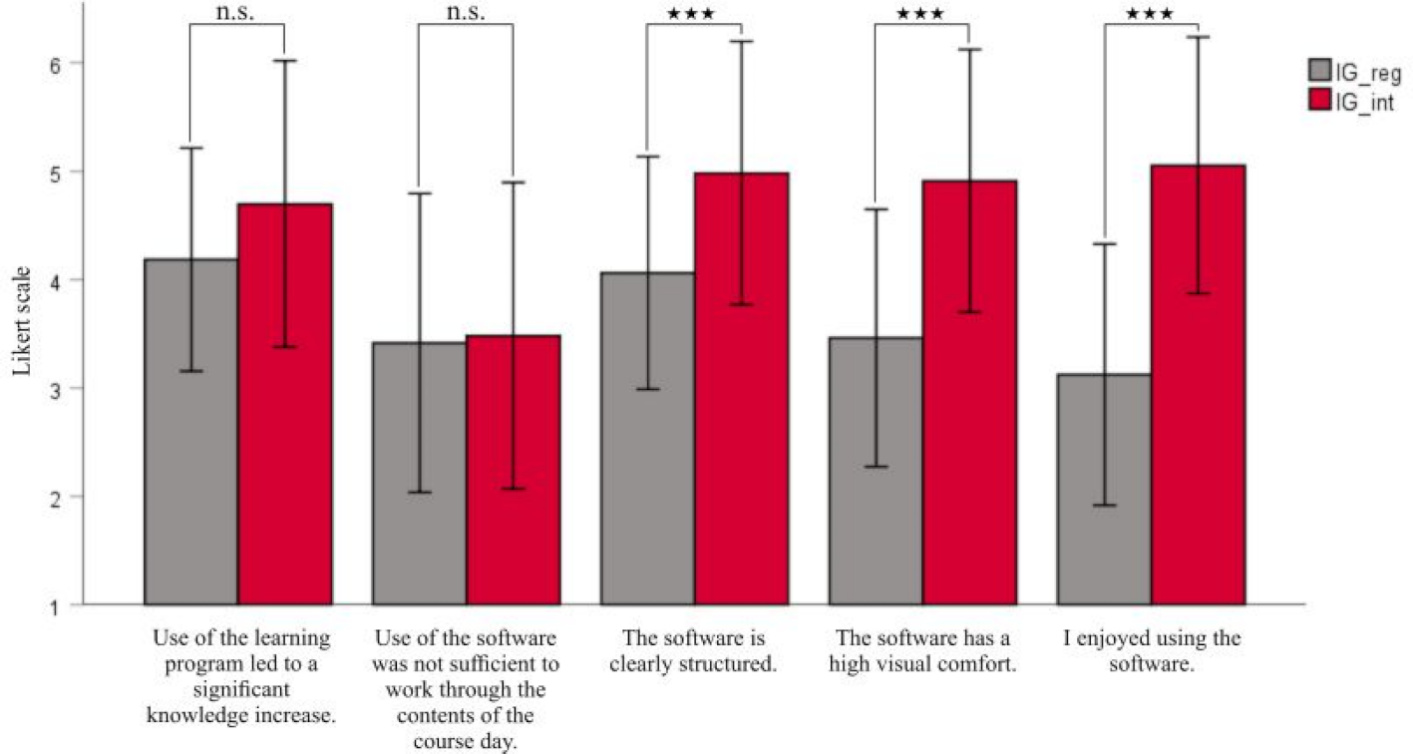
Results of the survey on usability and motivational factors. All students (n=121) of IG_reg (n=65) and IG_int (n=56) answered questions on various items about the user-friendliness and motivational aspects on an endpoint-based Likert scale from 1 (“Strongly disagree”) to 6 (“Strongly agree”). Inductive statistics used are outlined in 3.2. Tables 4 and 5 show the exact mean values and standard deviations as well as further items on the measurement of motivational factors and usability. ***p<.001, ns=not significant.

Students gave a positive assessment of the new software. There was a significant difference in the evaluation of motivation-relevant factors and usability between IG_int and IG_reg (*F*(7, 113)=12.48, *p*<.001, partial η^2^=.436). A separate ANOVA was carried out for every dependent variable. ANOVAs were conducted at an alpha level of .007. The interactive software was rated significantly higher regarding visual comfort (*F*(1, 119)=44.04, *p*<.001, partial η^2^=.270) and clear layout (*F*(1, 119)=19.61, *p*<.001, partial η^2^=.141) as compared to the traditional program (see figure 3 (Fig. 3)). The item “I enjoyed using the software” was also rated significantly better in IG_int than in IG_reg (*F*(1, 119)=78.55, *p*<.001, partial η^2^=.398). Regarding completeness of the histology contents (*F*(1, 119)=0.07, *p*=.793, partial η^2^=.001) and the remaining items, there was no significant difference between the two software versions (see table 4 (Tab. 4) and table 5 (Tab. 5)).

## 4. Discussion

### 4.1. Influence of the software versions

#### 4.1.1. Influence on the learning success

Our study showed that students achieved a significant increase in their learning success using the interactive software as compared to those students who had learned without any the two software versions. These observations were confirmed by studies in the subject Anatomy where the (additional) use of an e-learning module resulted in an increased learning success in the preclinical phase of medical education [28], [29]. Moreover, knowledge acquisition can be improved through the use of interactive elements [20]. Our comparison of both software versions with the CG confirmed that interactive elements can play a key role in the learning process.

In addition, there was no significant difference in the learning success between both software versions. This means that the interactive software is equivalent to the existing learning program – despite the superior rating of motivational factors. Moreover, this suggests that the histology contents of the interactive program correspond to the contents of the official software. It should be noted that there was a large statistical dispersion in figure 2 (Fig. 2) and figure 3 (Fig. 3). This aspect will be discussed in 4.1.3.

##### 4.1.2. Evaluation of motivational factors and usability

The results of our study confirm that usability and motivational aspects were rated significantly better using the interactive software as compared to the existing learning program. Accordingly, motivation can be increased through the integration of interactive elements and use of the software in preparation of the practical training day. The item “I enjoyed using the software” demonstrated the direct influence of the application on the intrinsic motivation. These results were confirmed by the results of a study in the subject Biochemistry. Here, a significant increase of the motivation was observed with the inverted classroom method where medical students prepared for the lecture using instructional films and reviewed the acquired knowledge by means of comprehension questions [17].

In general, the demand for digital services and e-learning applications in medical education has been rising constantly for a number of years [3], [16], [31]. In this context, interactive formats also gain more and more importance [18]. The results of our study confirm that in the implementation of a web-based application, educational models should already be incorporated during the planning phase in order to increase the learning success and above all motivation in the learning process [5], [12], [13], [14].

##### 4.1.3.Limitations and solutions

The study was limited by the fact that the evaluation of the learning success was only performed based on a small number of questions per test (n=4). This was unavoidable due to the course of the practical training, but should be modified in future surveys. One idea would be to evaluate several chapters and develop a questionnaire with a main focus on the quantification of knowledge acquisition. A critical point, however, is that students knew during the course to which study group they have been assigned. Another point relates to the CG that can only be formed in the assessment phase due to the self-reported learning behaviour. This learning behaviour might be attributable to a missing affinity towards electronic teaching media. Thus, evaluation might have been performed by participants with a particularly high intrinsic motivation. Furthermore, it should be taken into consideration that IG_int had more time to learn the contents due to the additional quiz questions – a factor which might be correlated with the learning success. Different learning periods within the groups and differences regarding the motivation could have influenced the statistical dispersion of the mean values. These factors should be investigated in a subsequent study and additional learning materials the individual students used identified. However, the variety of additional learning aids and subjective learning behaviour might impair a reliable quantification.

#### 4.2. Other digital services by comparison

Frequently used and popular digital medicine related learning aids are commercial platforms like Viamedici.Thieme.de, Medi-Learn.de, and Amboss.com. These platforms act as result- and exam-oriented applications neglecting certain aspects, such as intrinsic motivation that is important for learning. In this context, the problem area of limited validity of internet-related information should be mentioned [11]. The development of individual learning applications by medical institutes and specially trained employees as well as the implementation of suitable portals might provide an adequate solution [11]. In future, the design of software products with interactive elements and on the basis of learning theory models should therefore rather be entrusted to university teachers than commercial service providers. However, as only a few universities have an extensive quality management for e-learning programs in human medicine, such a tool should be introduced to be able to evaluate the quality of (digital) teaching platforms on a long-term basis [1]. In view of the digital change taking place at universities, framework conditions and funding opportunities should be adapted accordingly [9], [23]. 

## 5. Conclusions and outlook

In the coming semesters, further studies should be conducted in order to evaluate the efficiency of our software and user behaviour on a long-term basis.

An interactive optional online teaching service of the Philipps University Marburg in the subject Human Genetics was particularly well received by students in the clinical study phase [25]. As our program includes “Microscopic anatomy” and thus covers a subject of the preclinical phase incorporating diverse quiz types, the influence of the software should be further evaluated. Our findings have further shown that a subsequent evaluation study should be performed against the background of a long-term use.

Upon request and within the scope of future projects, transferability to other subjects of the preclinical and clinical study phase should be possible without any problem. As various contents can be integrated into the software, the e-learning service of curricular and non-curricular lectures can easily be extended.

## Funding

The project was supported by the Medical Faculty of the Goethe University Frankfurt within the scope of an application for funding of a teaching improvement project (Antrag auf Förderung eines Projektes zur Lehrverbesserung) (Decision S 59/2017).

## Acknowledgements

We thank Farid Theune and Wolfgang Gottlieb for their contributions in the development of the learning software “Histologie für Mediziner”.

## Competing interests

The authors declare that they have no competing interests. 

## Supplementary Material

Development of the software “Histologie für Mediziner” for medical histology courses: Sample learning unit

Development of the software “Histologie für Mediziner” for medical histology courses: Sample quiz questions

Further details on the development of the software “Histologie für Mediziner” for medical histology courses

Usability and motivation: Details on the questionnaire

## Figures and Tables

**Table 1:**
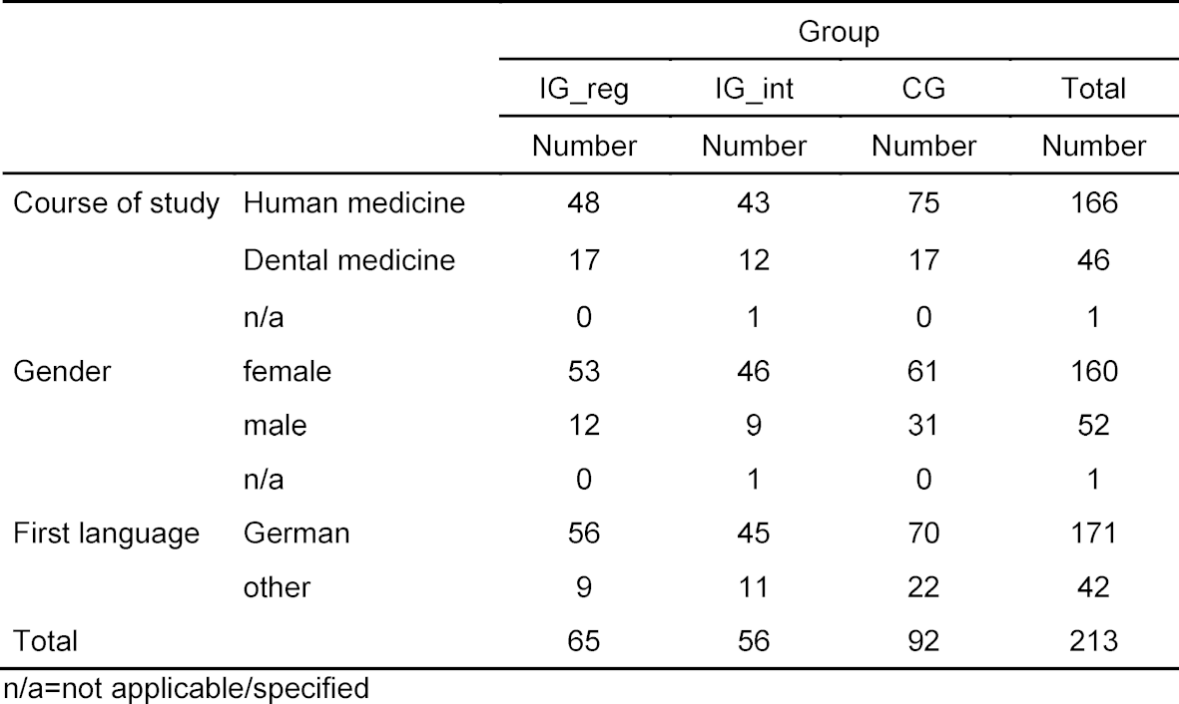
Number and distribution of students within the three groups

**Table 2:**
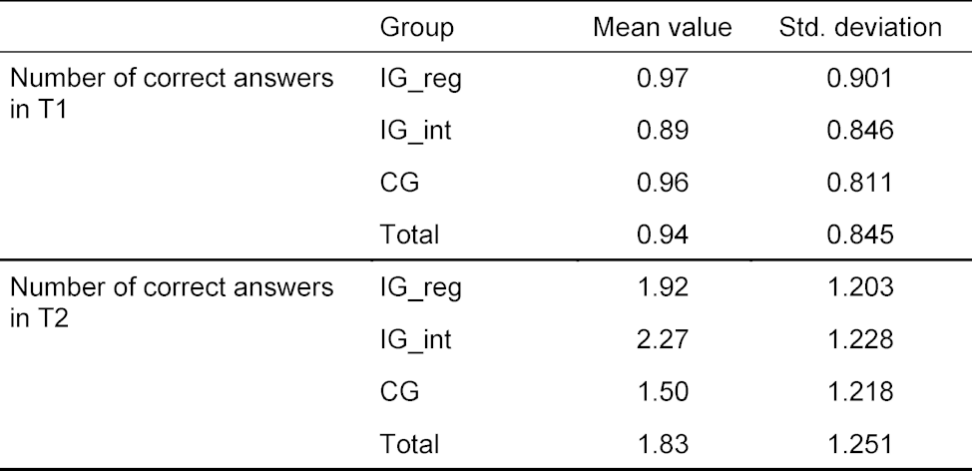
Descriptive depiction of the results of the knowledge test. Study participants (n=213) were assigned to either IG_reg (n=65), IG_int (n=56), or CG (n=92). The number of correct answers was measured at day one (T1) and week two (T2). In every test, a maximum of four points could be achieved.

**Table 3:**
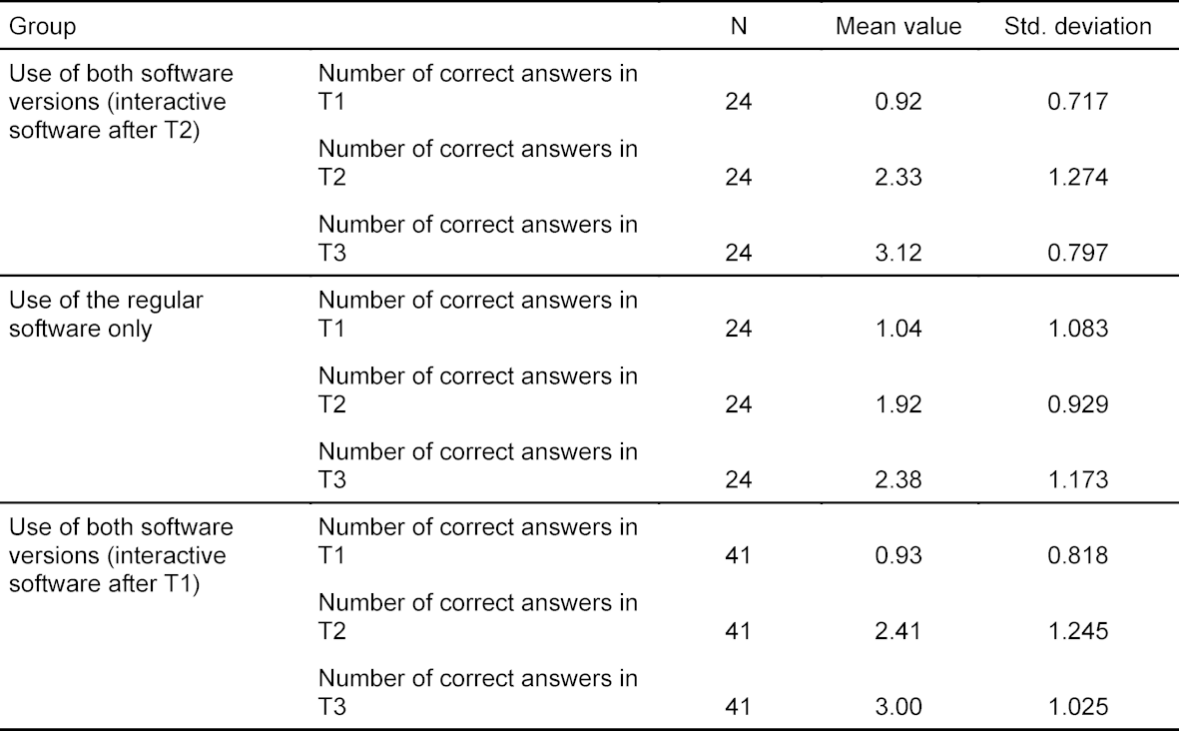
Results of the knowledge tests. The number of correct answers was measured at day one (T1), week two (T2), and week five (T3). In every test, a maximum of four points could be achieved. Between T2 and T3 the students were free to choose with which software version they wanted to learn. Due to the resulting small group size of the different subgroups, data are presented using descriptive statistics.

**Table 4:**
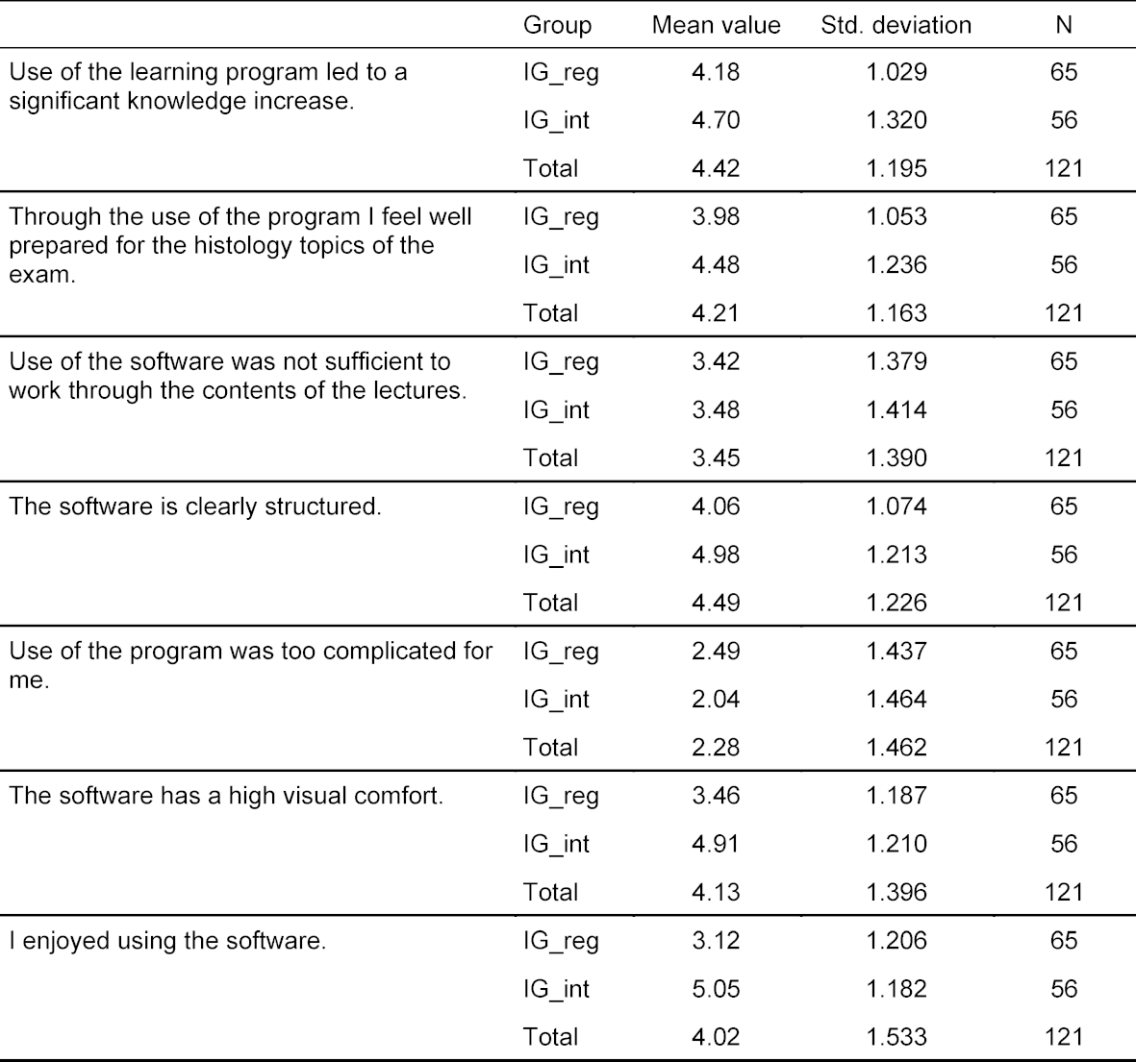
Descriptive depiction of the results of the usability and motivation survey. Evaluation was performed using an endpoint-based 6 stage Likert scale from 1 (“Strongly disagree”) to 6 (“Strongly agree”).

**Table 5:**
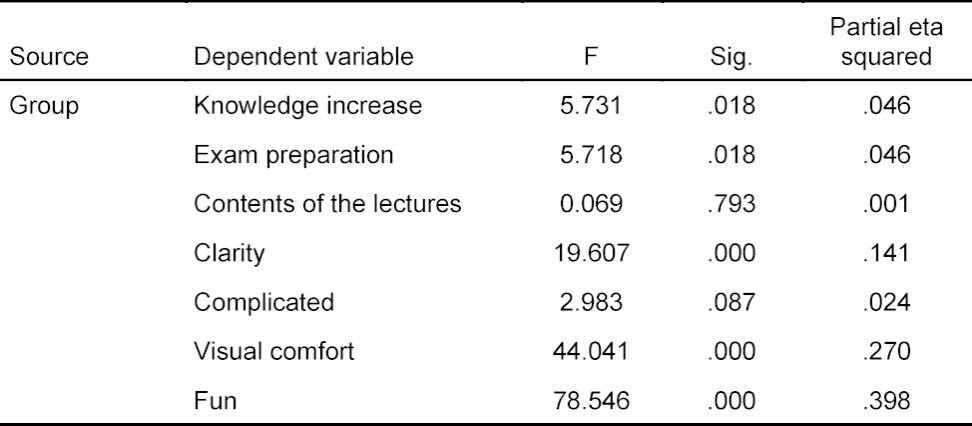
Statistics on the usability and motivation survey (df group=1, df error=119), tests of between-subjects effects
