# Supplementary material for: Development of an interactive e-learning software “Histologie für Mediziner” for medical histology courses and its overall impact on learning outcomes and motivation
Source: GMS J Med Educ. 2020 Apr 15;37(3):Doc35. doi: 10.3205/zma001328 (PMC7291388; doi:10.3205/zma001328)
Supplement: Development of the software “Histologie für Mediziner” for medical histology courses: Sample learning unit [file JME-37-35-s-001.pdf]

Attachment 1: Development of the software “Histologie für Mediziner” for medical histology courses: Sample learning unit

A

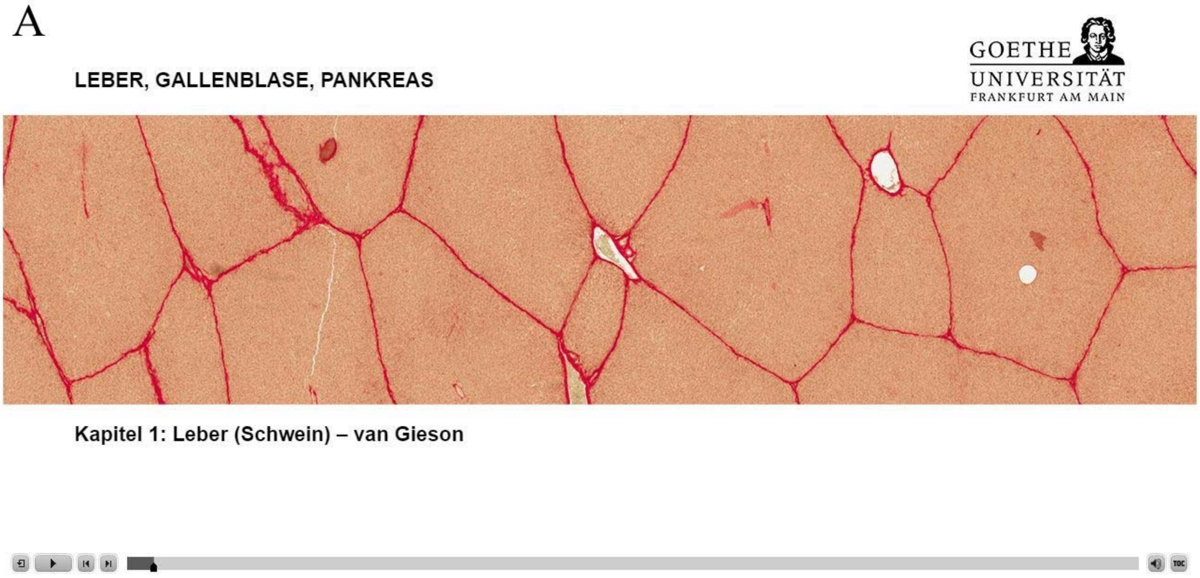

B

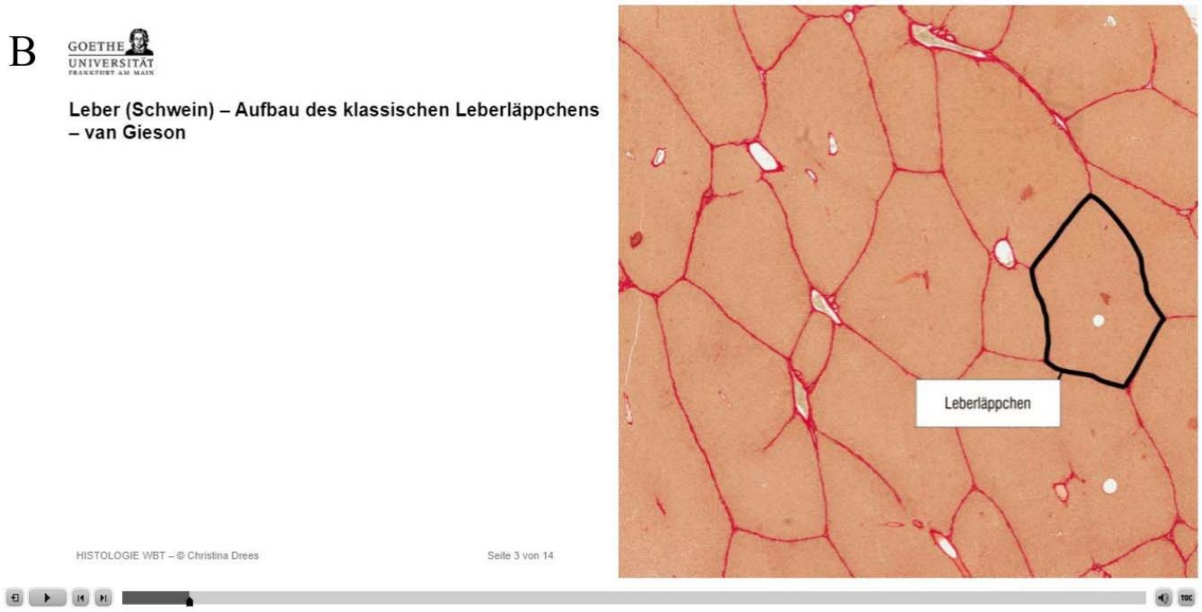

Attachment 1 to: *Drees C, Ghebremedhin E, Hansen M. Development of an interactive e-learning software “Histologie für Mediziner” for medical histology courses and its overall impact on learning outcomes and motivation. GMS J Med Educ. 2020;37(3):Doc35. DOI: 10.3205/zma001328.*
Online verfügbar unter: https://www.emgs.de/en/jounals/zma/2020-37/zma001328.shtml

C

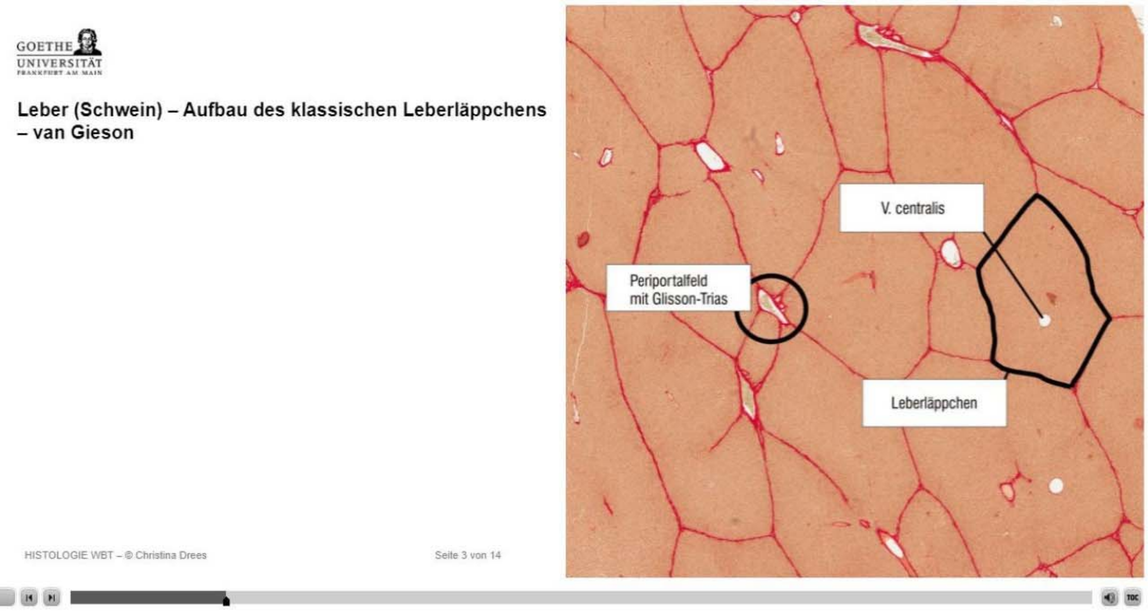

D

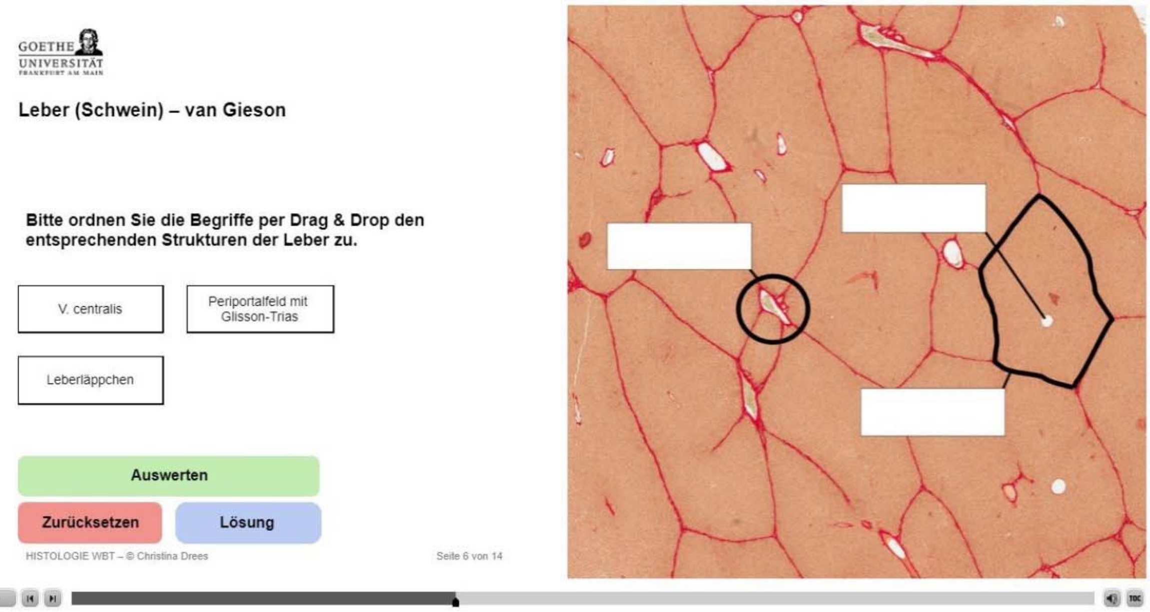

Attachment 1 to: *Drees C, Ghebremedhin E, Hansen M. Development of an interactive e-learning software “Histologie für Mediziner” for medical histology courses and its overall impact on learning outcomes and motivation. GMS J Med Educ. 2020;37(3):Doc35. DOI: 10.3205/zma001328.*
Online verfügbar unter: https://www.emgs.de/en/jounals/zma/2020-37/zma001328.shtml

Figure 1.1: Exemplary design of the LU “Liver, gall bladder, pancreas”. A-C. The LU of the new software contains multimedia and interactive elements. Contents are presented in spoken form while the corresponding terms are displayed simultaneously. D. In every LU, the students have to answer a quiz question after each section. Students can review the acquired knowledge by means of the drag-and-drop question shown here.

Translation (German-English):

- Leber, Gallenblase Pankreas = Liver, gall bladder, pancreas
- Kapitel 1: Leber (Schwein) – van Gieson = Chapter 1: Liver (porcine) – van Gieson
- Leber (Schwein) – Aufbau des klassischen Leberläppchens – van Gieson = Liver (porcine) – Classical structure of hepatic lobules – van Gieson
- Leberläppchen = Hepatic lobule
- V. centralis = Central vein
- Periportalfeld mit Glisson-Trias = Periportal space with Glisson's triad
- Bitte ordnen Sie die Begriffe per Drag & Drop den entsprechenden Strukturen der Leber zu. = Please assign the designation to the respective structure by drag & drop.
- Auswerten = Assess
- Zurücksetzen = Reset
- Lösung = Solution

Attachment 1 to: *Drees C, Ghebremedhin E, Hansen M. Development of an interactive e-learning software “Histologie für Mediziner” for medical histology courses and its overall impact on learning outcomes and motivation. GMS J Med Educ. 2020;37(3):Doc35. DOI: 10.3205/zma001328.*
Online verfügbar unter: https://www.emgs.de/en/jounals/zma/2020-37/zma001328.shtml
