# Supplementary material for: Development of an interactive e-learning software “Histologie für Mediziner” for medical histology courses and its overall impact on learning outcomes and motivation
Source: GMS J Med Educ. 2020 Apr 15;37(3):Doc35. doi: 10.3205/zma001328 (PMC7291388; doi:10.3205/zma001328)
Supplement: Development of the software “Histologie für Mediziner” for medical histology courses: Sample quiz questions [file JME-37-35-s-002.pdf]

Attachment 2: Development of the software “Histologie für Mediziner” for medical histology courses: Sample quiz questions

A

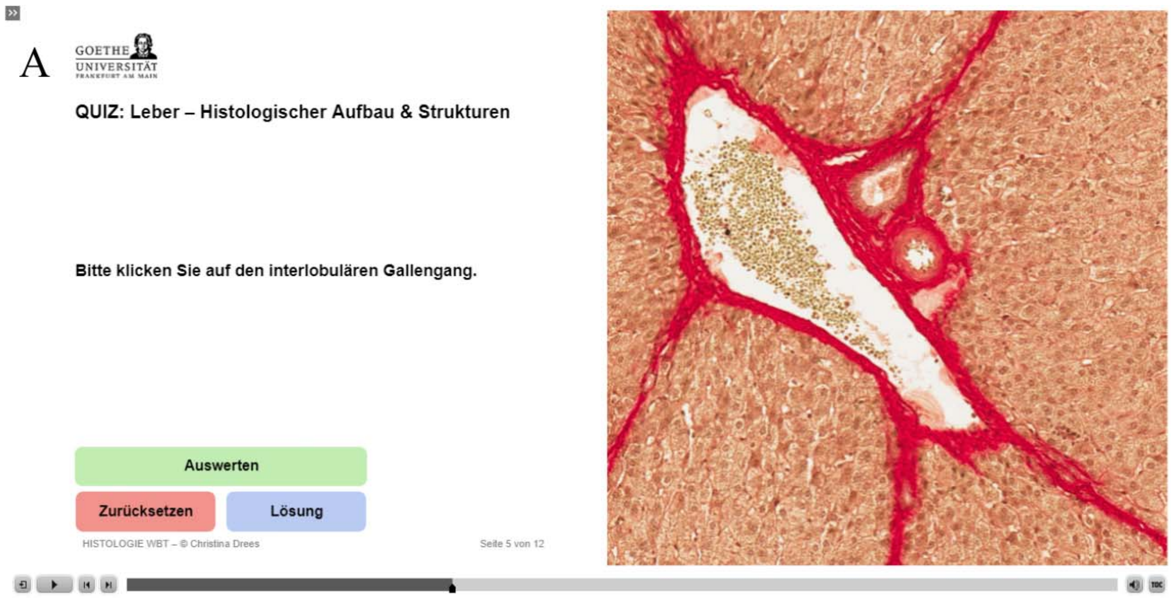

B

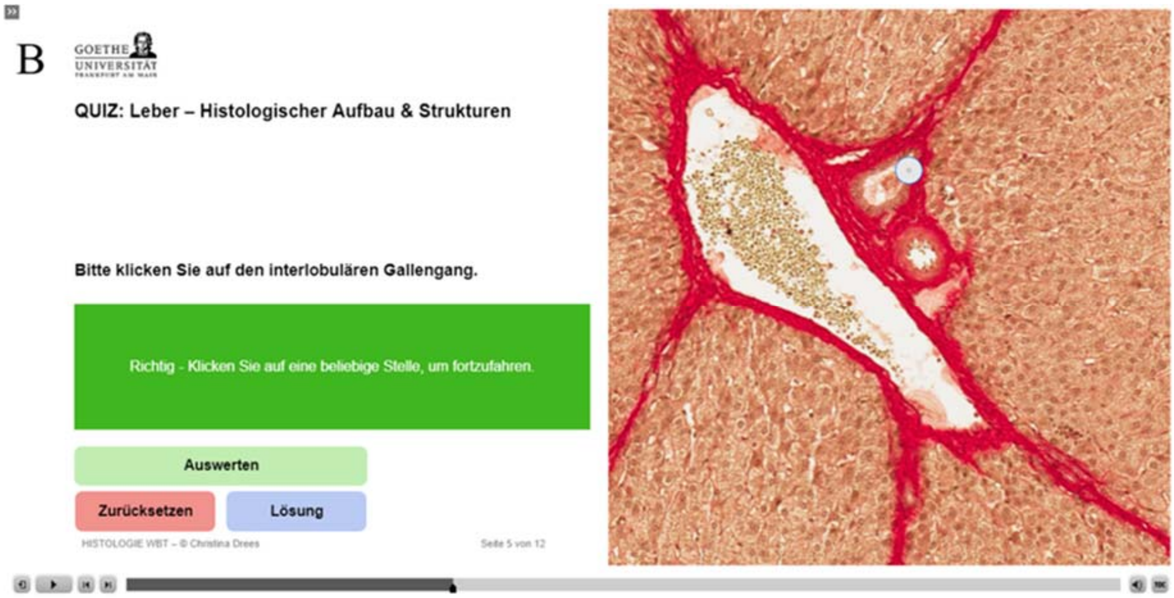

Attachment 2 to: *Drees C, Ghebremedhin E, Hansen M. Development of an interactive e-learning software “Histologie für Mediziner” for medical histology courses and its overall impact on learning outcomes and motivation. GMS J Med Educ. 2020;37(3):Doc35. DOI: 10.3205/zma001328.*
Online verfügbar unter: https://www.emgs.de/en/jounals/zma/2020-37/zma001328.shtml

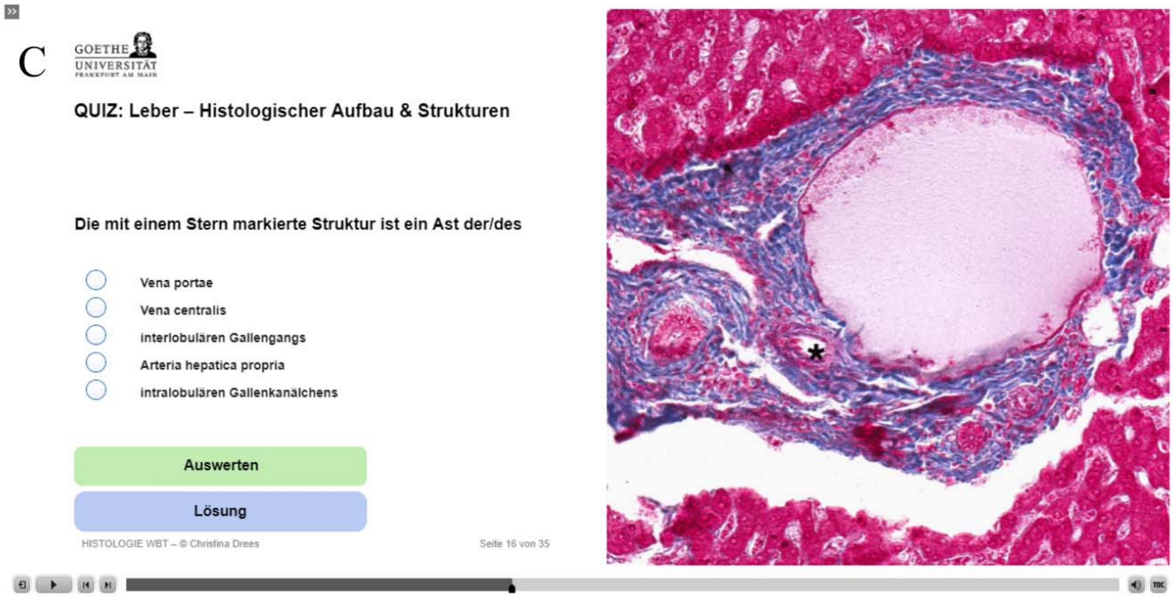

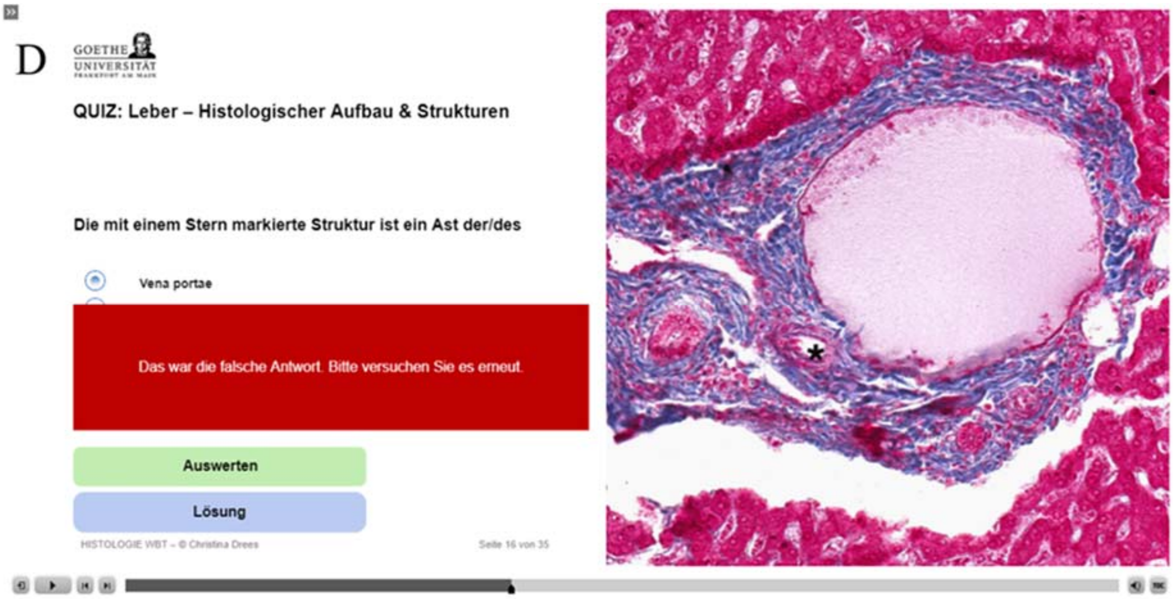

Attachment 2 to: *Drees C, Ghebremedhin E, Hansen M. Development of an interactive e-learning software “Histologie für Mediziner” for medical histology courses and its overall impact on learning outcomes and motivation. GMS J Med Educ. 2020;37(3):Doc35. DOI: 10.3205/zma001328.*
Online verfügbar unter: https://www.emgs.de/en/jounals/zma/2020-37/zma001328.shtml

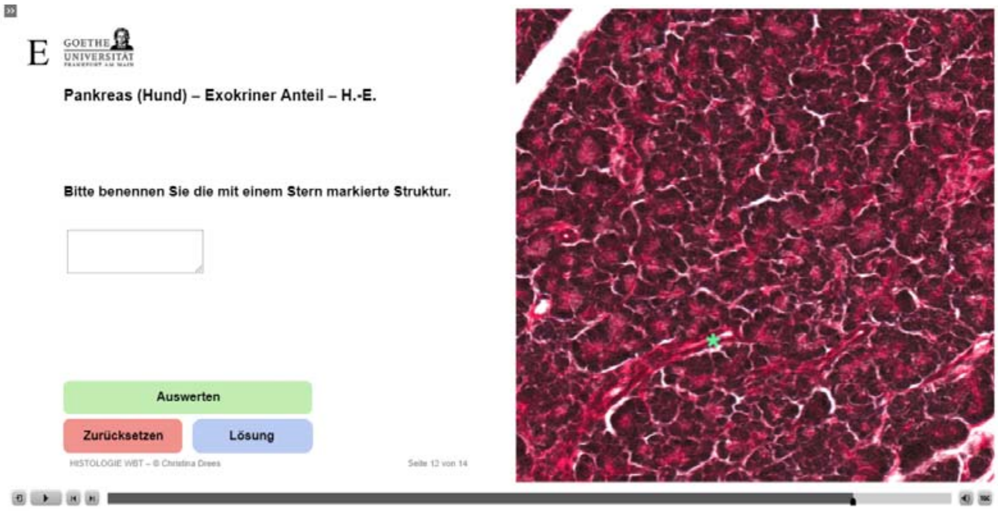

Figure 2.1: Exemplary depiction of the different quiz types. A. With hotspot questions, knowledge of structures is evaluated. Students can click on them. B. As soon as the corresponding structure was identified and marked, the student receives a direct, positive feedback. C. Multiple choice questions also enable the correct identification of histological structures. D. In case of a wrong answer, the student receives an audiovisual feedback, and the question can be answered again. E. Short answers provide the opportunity to test the active knowledge.

Translation (German-English):

- Quiz: Leber – Histologischer Aufbau & Strukturen = Quiz: Liver – histological composition & structures
- Bitte klicken Sie auf den interlobulären Gallengang. = Please click on the interlobular bile duct.
- Richtig – Klicken Sie auf eine beliebige Stelle, um fortzufahren. = Correct – Click on any position to continue.
- Die mit einem Stern markierte Struktur ist ein Ast der/des = The structure marked with an asterisk is a branch of the
- Vena portae = Portal vein
- Vena centralis = Central vein
- Interlobulären Gallengangs = Interlobular bile duct
- Arteria hepatica propria = Proper hepatic artery
- Intralobulären Gallenkanälchens = Intralobular bile duct
- Das war die falsche Antwort. Bitte versuchen Sie es erneut. = This was the wrong answer. Please try again.
- Pankreas (Hund) – Exokriner Anteil – H.-E. = Pancreas (canine) – exocrine part – H.-E.
- Bitte benennen Sie die mit einem Stern markierte Struktur. = Please name the structure marked with an asterisk.
- Auswerten = Assess
- Zurücksetzen = Reset
- Lösung = Solution
