# Supplementary material for: Development of an interactive e-learning software “Histologie für Mediziner” for medical histology courses and its overall impact on learning outcomes and motivation
Source: GMS J Med Educ. 2020 Apr 15;37(3):Doc35. doi: 10.3205/zma001328 (PMC7291388; doi:10.3205/zma001328)
Supplement: Further details on the development of the software “Histologie für Mediziner” for medical histology courses [file JME-37-35-s-003.pdf]

Attachment 3: Further details on the development of the software “Histologie für Mediziner” for medical histology courses

Spoken texts were recorded and mastered in collaboration with Wolfgang Gottlieb and Farid Theune at the Lotte Lindenberg sound studio in Frankfurt/Main.

Sounds and music were recorded at the JMW studio in Frankfurt/Main (see figure 3.1).

The following persons were involved in the development of the software:

- Texts & image processing: Christina Drees
- Music & sounds: Christina Drees, Matthias Vatter
- Speech recording & mastering: Christina Drees, Farid Theune, Wolfgang Gottlieb
- Software development & design: Christina Drees, Farid Theune, Wolfgang Gottlieb

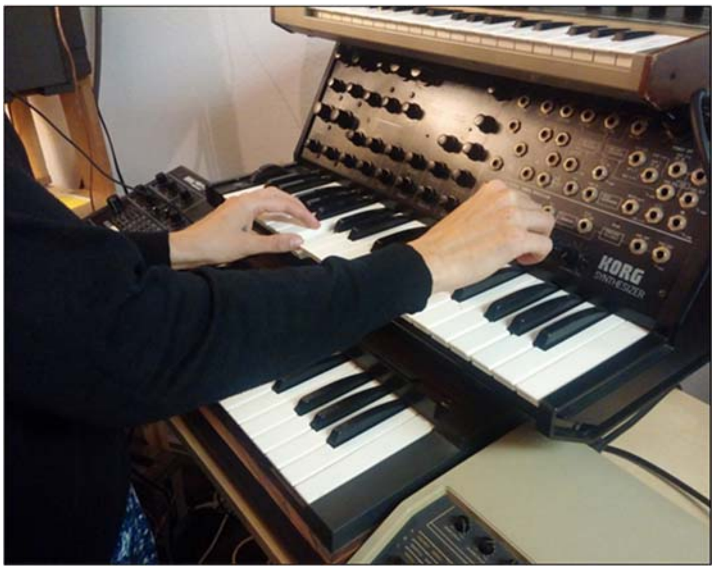

Figure 3.1: Sounds were recorded at the JMW-Studio in Frankfurt/Main, Germany.

Attachment 3 to: *Drees C, Ghebremedhin E, Hansen M. Development of an interactive e-learning software “Histologie für Mediziner” for medical histology courses and its overall impact on learning outcomes and motivation. GMS J Med Educ. 2020;37(3):Doc35. DOI: 10.3205/zma001328.*
Online verfügbar unter: https://www.emgs.de/en/jounals/zma/2020-37/zma001328.shtml
