# Supplementary material for: Development of an interactive e-learning software “Histologie für Mediziner” for medical histology courses and its overall impact on learning outcomes and motivation
Source: GMS J Med Educ. 2020 Apr 15;37(3):Doc35. doi: 10.3205/zma001328 (PMC7291388; doi:10.3205/zma001328)
Supplement: Usability and motivation: Details on the questionnaire [file JME-37-35-s-004.pdf]

Attachment 4: Usability and motivation: Details on the questionnaire

In addition to histology questions, questionnaire T2 contained seven different items to evaluate motivational factors and usability. Evaluation was performed using an endpoint-based 6 stage Likert scale from 1 (“Strongly disagree”) to 6 (“Strongly agree”).

T2 included the following items:

1. Use of the learning program led to a significant knowledge increase.
2. Through the use of the program I feel well prepared for the histology topics of the exam.
3. Use of the software was not sufficient to work through the contents of the course day.
4. The software is clearly structured.
5. Use of the program was too complicated for me.
6. The software has a high visual comfort.
7. I enjoyed using the software.

Attachment 4 to: *Drees C, Ghebremedhin E, Hansen M. Development of an interactive e-learning software “Histologie für Mediziner” for medical histology courses and its overall impact on learning outcomes and motivation. GMS J Med Educ. 2020;37(3):Doc35. DOI: 10.3205/zma001328.*
Online verfügbar unter: https://www.emgs.de/en/jounals/zma/2020-37/zma001328.shtml
